# Supplementary figures and images for: Targeted protein degradation by KLHDC2 ligands identified by high-throughput screening
Source: eLife. 2025 Jun 16;14:RP106844. doi: 10.7554/eLife.106844 (PMC12169847; doi:10.7554/eLife.106844)

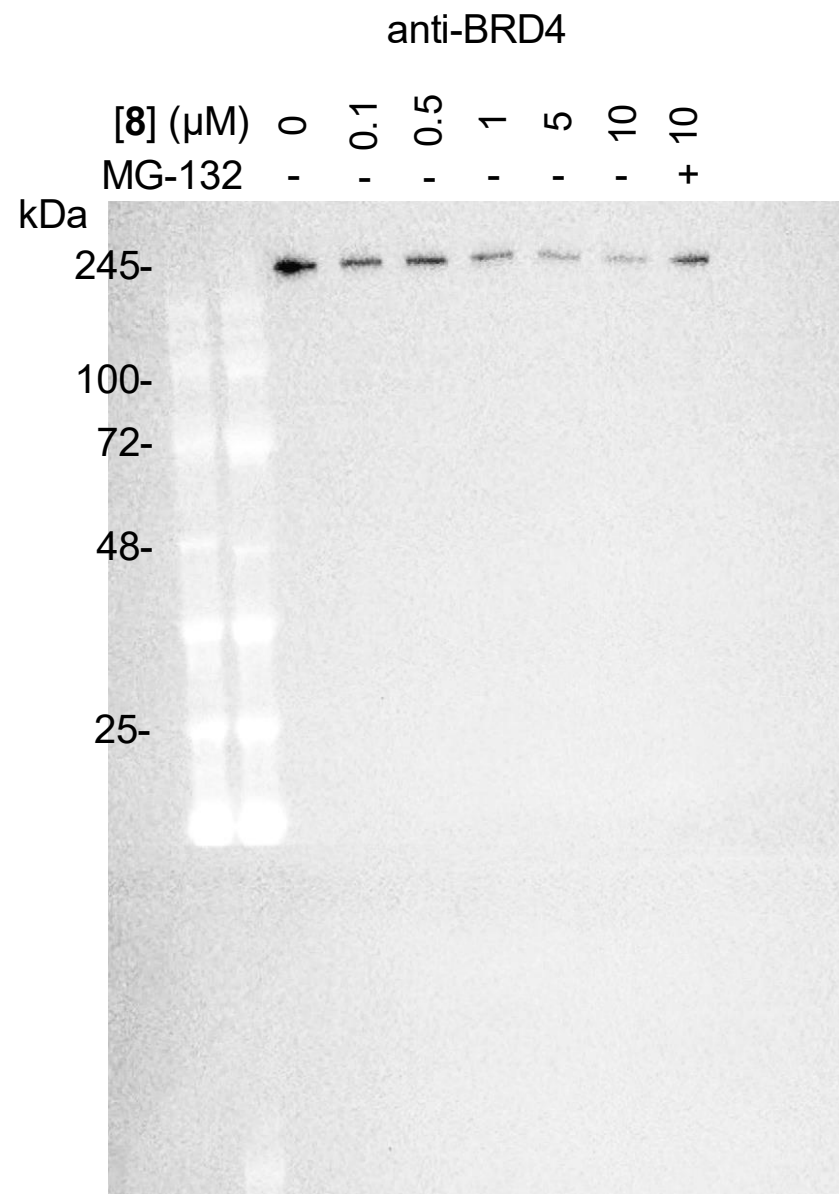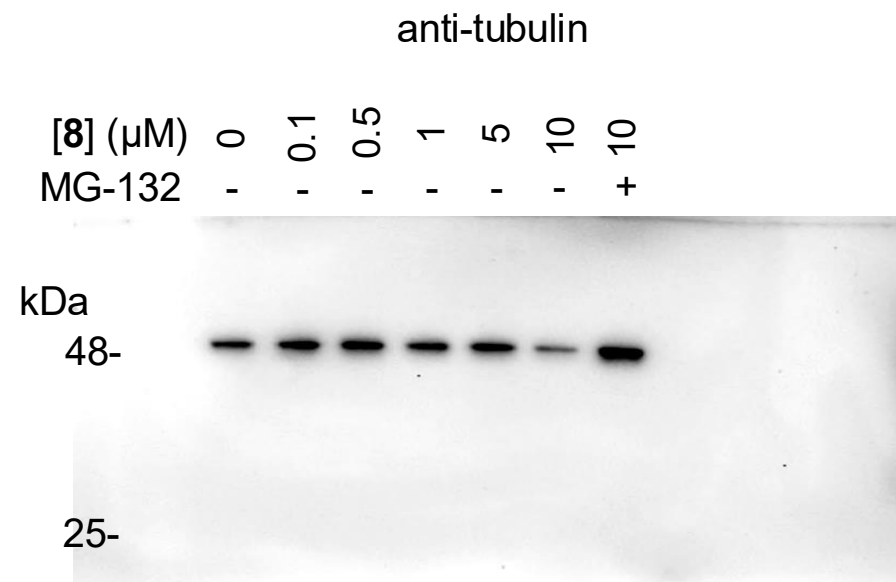

Supplement: Figure 4—source data 2. [file elife-106844-fig4-data2.zip › Figure 4E - uncropped blots.pdf]

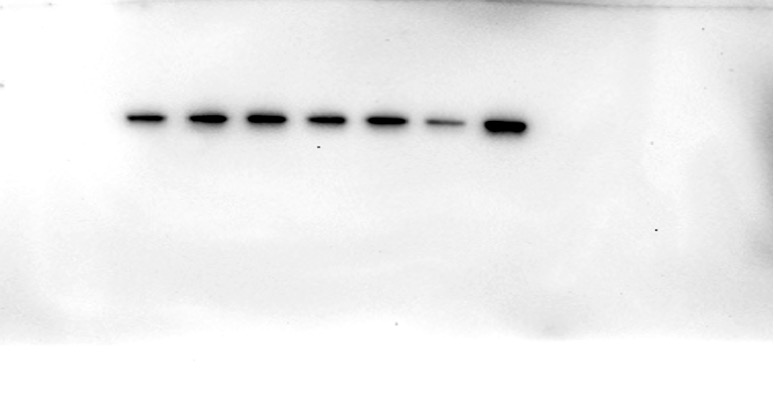

Supplement: Figure 4—source data 3. [file elife-106844-fig4-data3.zip › Figure 4E - tubulin.tiff]

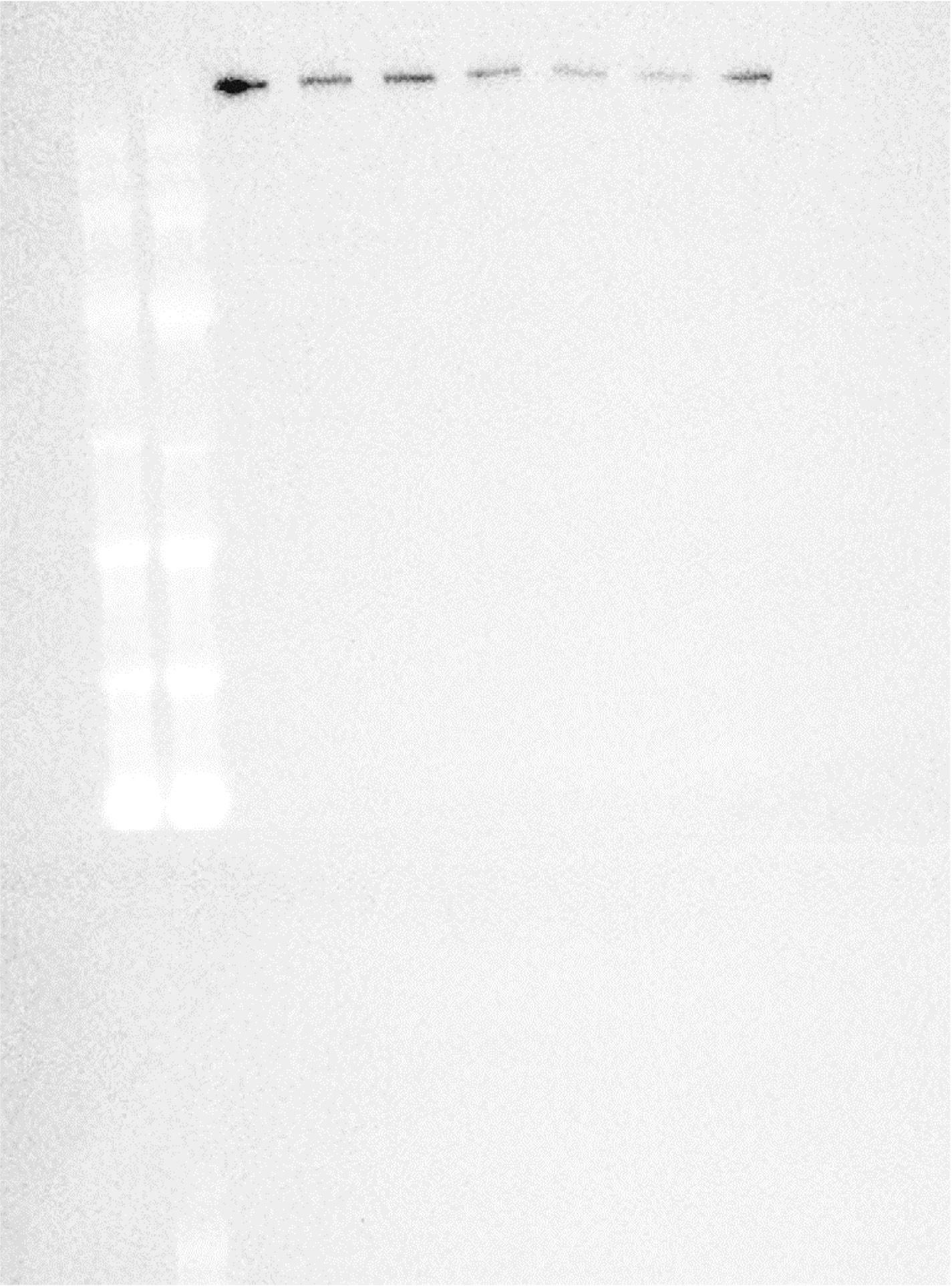

Supplement: Figure 4—source data 3. [file elife-106844-fig4-data3.zip › Figure 4E - BRD4.tiff]
